# CPP-Assisted Intracellular Drug Delivery, What Is Next?

**DOI:** 10.3390/ijms17111892

**Published:** 2016-11-14

**Authors:** Junxiao Ye, Ergang Liu, Zhili Yu, Xing Pei, Sunhui Chen, Pengwei Zhang, Meong-Cheol Shin, Junbo Gong, Huining He, Victor C. Yang

**Affiliations:** 1 Collaborative Innovation Center of Chemical Science and Chemical Engineering, School of Chemical Engineering and Technology, Tianjin University, Tianjin 300072, China; jasmineye2014@163.com (J.Y.); liuergang@hotmail.com (E.L.); 15122802060@126.com (P.Z.); junbo_gong@tju.edu.cn (J.G.); 2 Tianjin Key Laboratory on Technologies Enabling, Development of Clinical Therapeutics and Diagnostics (Theranostics), School of Pharmacy, Tianjin Medical University, Tianjin 300070, China; yuzhilinancy@163.com (Z.Y.); 13512232903@163.com (X.P.); annanchensunhui@foxmail.com (S.C.); 3 College of Pharmacy and Research Institute of Pharmaceutical Sciences, Gyeongsang National University, Jinjudaero, Jinju, Gyeongnam 660-751, Korea; shinmc@gnu.ac.kr; 4 Department of Pharmaceutical Sciences, College of Pharmacy, University of Michigan, Ann Arbor, MI 48109-1065, USA

**Keywords:** CPPs, intracellular delivery, pH and enzyme triggered drug delivery system

## Abstract

For the past 20 years, we have witnessed an unprecedented and, indeed, rather miraculous event of how cell-penetrating peptides (CPPs), the naturally originated penetrating enhancers, help overcome the membrane barrier that has hindered the access of bio-macromolecular compounds such as genes and proteins into cells, thereby denying their clinical potential to become potent anti-cancer drugs. By taking the advantage of the unique cell-translocation property of these short peptides, various payloads of proteins, nucleic acids, or even nanoparticle-based carriers were delivered into all cell types with unparalleled efficiency. However, non-specific CPP-mediated cell penetration into normal tissues can lead to widespread organ distribution of the payloads, thereby reducing the therapeutic efficacy of the drug and at the same time increasing the drug-induced toxic effects. In view of these challenges, we present herein a review of the new designs of CPP-linked vehicles and strategies to achieve highly effective yet less toxic chemotherapy in combating tumor oncology.

## 1. Introduction

Nowadays, with the rapid development in molecular engineering, genomics, and nanotechnologies, a dramatically increased number of new drugs, such as short peptides, proteins monoclonal antibodies, antisense oligonucleotides, ribozyme and catalytic DNAs were developed for cancer therapy. However, success in administrating this bio-macromolecular therapeutics was hampered by their large molecular size and hydrophilic property, which prevented these agents to cross the cell membrane barriers. Thus, cellular delivery of these hydrophilic bio-macromolecular compounds is still the bottleneck for their successful applications [1,2,3]. At present, despite that many drug delivery strategies have been developed, such as using viral vectors, cationic polymers, nanocarriers, etc., these methods nevertheless often suffer their own shortcomings, especially in in vivo applications, such as high toxicity caused by the host immune system, lack of ability to reach the designated cellular and intracellular target sites, etc. [1,4,5,6]. Overall, the primary drawback of these intracellular delivery strategies lies in their need of an endocytic pathway to overcome the cell membrane barrier, rendering these macromolecular drugs unable to escape from the endosomes, thereby causing their ultimate degradation in the lysosomes [1,7,8,9]. Therefore, to satisfy the absolute need of intracellular delivery of these macromolecular drugs, novel carriers possessing low toxicity but high cell transduction efficiencies, especially the ability to directly deliver the payload into cytoplasm, nucleus or other specific organelles (e.g., lysosomes, mitochondria or even endoplasmic reticulum), become one of the Gordian knots to achieve organelle-targeted therapy. In this regard, cell penetrating peptides (CPPs) emerge as one of the most important entities in many drug delivery systems since last century [10,11,12,13,14].

For the past 20 years, we have witnessed an unprecedented and, indeed, rather miraculous event of how cell-penetrating peptides can assist bio-macromolecular compounds such as proteins and gene therapeutics to overcome the membrane barriers in achieving cellular internalization. The CPP-assisted intracellular delivery of large compounds was so overwhelmingly effective that these peptides [2,11,15,16,17,18,19,20,21,22] were given the unique name of “Trojan horse” in their associated delivery systems. In principle and practice, CPPs were shown to be capable of overcoming various types of barriers, such as the blood–brain barrier (BBB) [23,24], intestine wall [25,26], retina [16,27] neurons and even the skin [21,28]. By virtue of these cell penetrating peptides, various drug payloads were shown to gain access to the cytoplasmic compartment with extraordinary efficiency both in vitro and in vivo. In this review, we provide a succinct summary of recent findings related to CPP-assisted strategies in achieving highly effective yet less toxic intracellular delivery of proteins, nucleic acids and nanoparticles into specific sub-cellular organelles in the combat against tumor oncology.

## 2. Cell Penetrating Peptides (CPPs)-Assisted Strategies for Intracellular Drug Delivery

**Figure 1:**
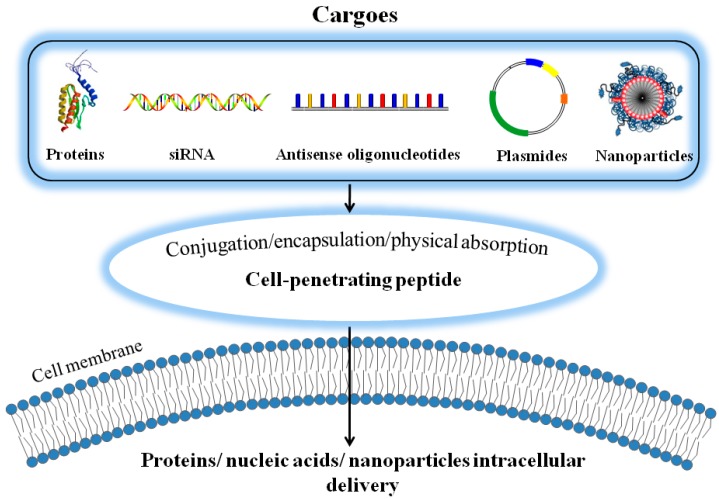
Cell penetrating peptide (CPP)-assisted strategies employed in achieving intracellular delivery of bio-macromolecular compounds and nano-carriers.

Since the first discovery of the TAT-class of CPPs in 1988 [29,30], these short sequence peptides were widely applied to enhance delivery of macromolecules into cells of different tissue/organ types. Subsequently, various classes of CPPs, including arginine/lysine-rich cationic peptides, LMWP, Penetratin and VP22 [15,24,31,32,33,34], as well as amphipathic peptides such as MAP, MPG, pep-1, etc., were also reported and utilized in either remarkable intracellular delivery strategies or the successful application in medical and biological field [17,19,25,26,27,35,36,37,38,39,40,41,42,43,44,45,46,47,48]. Via covalent linkage or non-covalent charge-associated aggregation, linkage, both in vitro and in vivo results demonstrated that CPPs could assist the delivery of virtually all types of molecular cargos including proteins [2,16,20,26,46,49,50,51,52,53,54,55], nucleic acids [33,35,36,56,57,58,59,60], and nano-carriers such as magnetic iron oxide nanoparticles (MION) [61,62,63,64,65,66,67,68] into cells. In general, the CPP-assisted drug delivery can be realized through three means: (1) via covalent conjugation of the drug molecule with a CPP; (2) encapsulation of the therapeutics into CPP-linked nano-carriers; and (3) by physical adsorption of the therapeutic agents with CPPs via charge complexation. Figure 1 illustrates such CPP-assisted strategies in achieving intracellular delivery of the drug payloads.

### 2.1. Covalent Conjugation of Therapeutic Agents with CPP

The commonly used strategy for CPP-assisted drug delivery is by covalent conjugation, either chemically or biologically, of CPPs with the therapeutic agents. In 1999, a recombinant TAT-β-galactosidase fusion protein that could cross through BBB following intraperitoneal injection was first reported by Dowdy and co-workers [23], which actually set up the tune to realize the milestone goal in pharmaceutical research in achieving intracellular delivery of protein drugs. Recently, we reported the successful synthesis of cell-permeable gelonin (Gel), a toxin that could inhibit protein translation in cells, with cationic CPPs (i.e., TAT and LMWP) via either chemical conjugation (CPP-Gel) or genetic recombinant method (rGel), to overcome the inability of gelonin to internalize cells [31]. Unlike native gelonin that could not enter cells to exert its cytotoxic effects, significant inhibition on tumor growth was observed in mice treated with CPP-Gel or rGel; suggesting that incorporation of CPP would dramatically enhance the cytotoxic activity of gelonin. On a different application, we also reported the formulation of homogenous, monomeric (1:1 ratio) LMWP-insulin covalent conjugate, which was capable of crossing the intestinal mucosal barrier, significantly augmenting the anti-diabetic effects of insulin [41,69,70].

### 2.2. Encapsulation of Therapeutic Agents into CPP-Linked Nano-Carriers

By taking the advantages that CPP-mediated cell translocation would not alter the structural and/or functional attributes of the cell membranes, Stroh et al. encapsulated quantum dots into TAT-modified micelles, which were taken up by mouse endothelial cells, thereby allowing tracking of these labeled cells in the tumor endothelium [71]. A variety of examples related to utilizing this delivery strategy, such as encapsulation of therapeutic protein (asparaginase) into red blood cells as well as drugs into CPP-linked nano-carriers were presented later in the subsequent sections.

### 2.3. Physical Adsorption of Therapeutic Agents with CPPs via Electrostatic Complexation

Another strategy for the CPPs-mediated drug delivery is by non-covalent physical adsorption of the anionic drugs (e.g., DNAs, and siRNAs) with the cationic CPPs via electrostatic interactions. In a recent study, we demonstrated the plausibility of this approach by physical adsorption of the anionic pSV-β-galactosidase plasmid with LMWP. Expression of β-galactosidase in animals was found to be significantly augmented with very minor cell cytotoxicity, when comparing with the use of cationic protamine or polyethyleneimine (PEI) as the β-galactosidase gene carrier [31]. Moreover, the LMWP/siRNA complex was also used for cancer therapy, as the fluorescent-tagged LMWP/siRNAs complexes were found to localize in the cytoplasm shortly after incubation with the carcinoma cells, resulting in a strong down-regulation of the target gene [72].

## 3. Mechanisms of the CPP-Mediated Intracellular Uptake

**Figure 2:**
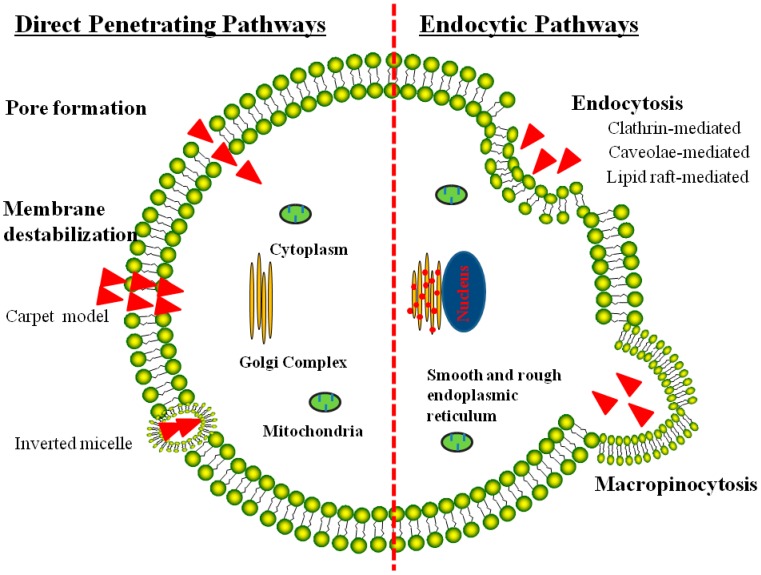
Mechanisms reported for the CPP-mediated intracellular drug delivery strategy.

The CPPs-mediated cell internalization was so overwhelming that it could not be matched by other conventional cell-entry methods such as the receptor-mediated endocytosis. Importantly, it was shown that CPPs-assisted cell internalization would not induce any perturbation or alteration of the cell membrane. Although the mechanisms of CPP-mediated cytoplasmic translocation are not fully understood yet, they nevertheless can be classified into two pathways: (1) direct translocation of CPPs across the cell membranes by electrostatic interaction with the negatively charged lipid bilayers or through hydrogen bonding [1,73,74]; and (2) energy-dependent macropinocytosis that is responsible for CPP-mediated intracellular delivery of large molecules and nanoparticles [75] (Figure 2). It was already confirmed that CPPs and their modified cargos could internalize cells via single or multiple endocytotic pathways [76], and then locate in different sub-cellular compartments such as cytoplasma or nucleus [1]. The variety of pathways appeared to correlate with the chemical and physical variability of the peptide sequences, CPP concentrations, and characteristics of the drug cargoes [77]. Nevertheless, the exact uptake mechanisms for the individual CPPs and CPP-modified conjugates still remain as a controversial issue. Many researchers also reported that uptake of CPPs including TAT, polyarginines, and penetratin were via endocytosis, which was similar to the internalization process of large CPP-fused molecular weight cargoes [78].

### 3.1. Direct Translocation of CPPs across Biological Membranes

Initial research reports indicated that translocation of the CPPs occurred even at low temperatures, suggesting the existence of an energy-independent uptake mechanism. Studies also demonstrated that CPPs prepared with d- or l-l amino acid enantiomers shared similar translocation efficiency, without the involvement of receptors in their cell internalization [79,80]. Based on these observations, several models were proposed to explain this direct cell translocation process, including the formation of “membrane pores” and membrane destabilization like the “inverted micelle model”, and the “carpet model” [5,81] (see the left panel in Figure 2). Subsequently, a few studies also demonstrated that translocation of CPPs across cell membrane was resulted from the artifacts caused by cell fixation, leading to suggestions of a full re-evaluation of the mechanisms involved in CPP-mediated cell translocation [82,83].

For the “converted micelle model”, it suggested an interaction of the CPP with the membrane, causing disturbance of the lipid bilayer and subsequently the formation of inverted hexagonal structures termed inverted micelles. In this model, the CPP was trapped in the hydrophilic environment of the micelle core, and further interaction between the membrane components with the CPP led to the destabilization of the formed micelles, releasing the CPP into the cytosolic compartment. This model was supported by NMR data on the interaction of the pAntp peptide with the cell membrane [14]. In another model involving the formation of “membrane pores”, translocations of CPPs or their associated conjugates across the membrane was achieved by the formation of transient pores caused by the insertion of the CPP into the membrane in a ring-shape structure [84,85,86]. With regard to the “carpet model”, CPPs or their associated conjugates occurred via a transient destabilization of the biological membrane, which was induced by the extensive association of CPP to the membrane and subsequently the event of phospholipid reorganization [84,85]. It should be noted that all of these three models appeared to be reasonable to account for the internalization event of large molecules across the impermeable cell membrane. In addition, these models all required the presence of amphipathic alpha-helix secondary structures, a feature that was indeed shared by many of the CPPs. Nevertheless, translocation of large molecules via these mechanisms would imply an extensive destabilization of the cell membrane, which was not truly compatible with the low cytotoxicity normally observed during CPP-mediated cell translocation. Based on these arguments, an alternative mechanism deems to play a role in CPP-mediated translocation, especially when CPP was conjugated with macromolecular cargoes.

### 3.2. Endocytosis Pathway for CPP-Mediated Cell Internalization

Comprehensive evaluation studies performed in live cells provided evidences that aside from the already described endocytosis-independent mechanisms, and cell translocation of CPPs via several endocytotic pathways, such as caveolae-mediated endocytosis [87,88], clathrin-mediated endocytosis, lipid raft-mediated endocytosis [89] and macropinocytosis [75,78] played important roles in CPP-mediated cell internalization. When CPP conjugate to large molecular weight cargos, like nano-carriers or large proteins large than 30 kDa, were delivered intracellularly through the endocytic pathways, however, the endocytic uptake pathways for CPPs mediated delivery was strongly depended on its attached cargoes [90]. Take TAT for example, once TAT was conjugated to protein, the conjugate passed the membrane via lipid raft-mediated endocytosis [88], while when TAT was conjugated to a fluorophore was taken up by the clathrin-dependent endocytosis [89]. Moreover, uptaken by the endocytosis pathways, the bioavailability and activity of the CPPs and its cargo was greatly restricted by the time needed for arrival at the target and storage of the internalized CPP species and its cargo in endosomes or lysosomes. Because the delivered bioactive molecules targeted outside endocytic vesicles (e.g., nucleus, mitochondrion), must escaped from endosomal vesicles before trafficking back to the plasma membrane for recycling or fusion with lysosomes. The CPPs mediated intracellular delivery of the large biomolecules and/or the nanoparticles can also be proceeded via energy-dependent macropinocytosis pathway, with subsequent escape from the endosome into the cytoplasm [75]. Another study suggested that TAT fusion proteins enter cells via the endosomal pathway, circumvent lysosomal degradation, and then sequester in the periphery of the nucleus [91]. In conclusion, CPPs mediated intracellular delivery of macromolecules and nanoparticles proceed via the energy-dependent endocytosis pathways and/or the macropinocytosis pathways, with subsequent escape from endosome into the cell cytoplasm, but the individual CPPs or CPPs-conjugated small molecules drugs penetrated cells via electrostatic interactions and hydrogen bonding and did not seem to depend on energy [91].

## 4. Technical Limitations Concerning CPPs-Assisted Intracellular Delivery

Despite their unprecedented efficiency in delivering therapeutic cargos into cells, clinical applications of CPP-mediated strategies still face several critical drawbacks and limitations. First, CPPs were all prone to enzymatic degradation by the circulating plasma proteases and thus need to be sterically protected [92,93] before reaching the target. To this regard, the use of protease-resistant CPPs consisting of d-form of amino acids deems to be an elegant strategy to resolve this problem [94]. Secondly, CPPs lack selectivity towards cells that they come in contact with, thereby triggering drug-induced toxic effects towards normal tissues. To prevail over this non-selectivity issue, an approach was suggested by incorporating the CPP molecules onto the so-called “smart” nano-carrier delivery platform, of which the non-specific cell-penetration functions of CPPs were sterically shielded by PEG chains attached on the surface of the carrier via stimulus-sensitive bonds. During the phase of tumor targeting, CPPs would remain inactive. However, once accumulated at the tumor target, the protective PEG moieties on the surface of the carrier would be automatically detached in response to the changes of environmental conditions around the tumor, revealing these CPP moieties of their potent cell-penetrating functions at the tumor target [21,95,96,97]. Changes in local environmental conditions typically seen in cancers, infarcts and inflammations are reduced pH, elevated temperature, and the presence of overexpressed matrix metalloproteinase activities (MMPs). In addition, external triggers such as heat, radiation, ultrasound, radiofrequencies and magnetic fields can also be used to inactivate PEG protection.

## 5. Stimulus-Responsive “Smart” Systems for CPP-Mediated Cancer Therapy

### 5.1. Prodrug-Based, CPP-Mediated Smart Drug Delivery Systems

**Figure 3:**
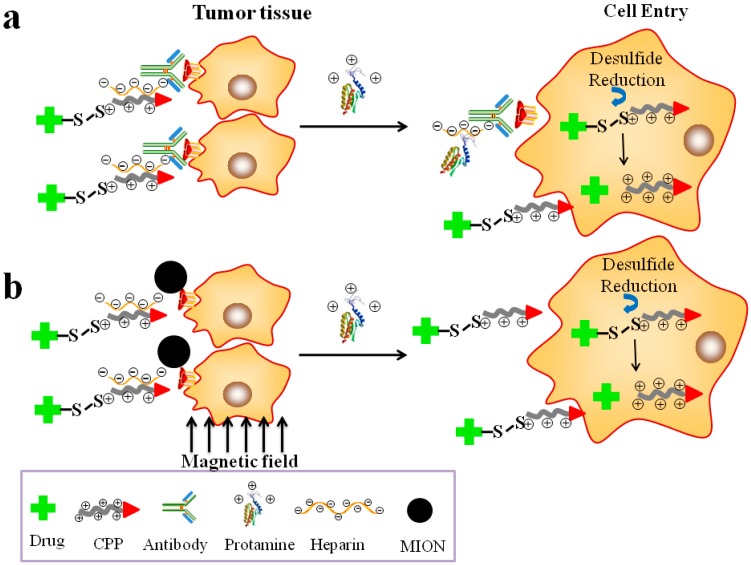
Illustration of the: (**a**) antibody-guided; and (**b**) Magnetic iron oxide nanoparticles (MION)-involved targeted drug delivery system (Reproduced according to [98,102]).

In order to safe control this CPP “Trojan Horse”-led intracellular drug delivery, “smart” systems were developed using the heparin/protamine-regulated prodrug protection/deprotection strategy [17,31,52]. This system was made up of two major components: (a) a targeting compartment that consisted of a heparin-linked tumor-targeting unit such as an antibody (Figure 3a) or the magnetic iron oxide nanoparticle (MION) carrier (Figure 3b); and (b) a drug compartment composed of a covalent conjugate of the polycationic CPPs (e.g., TAT and LMWP) and macromolecular drugs (e.g., protein and siRNA) in achieving potent intra-tumoral drug therapy [66,67,68,98,99]. The charge-based prodrug behavior was self-assembled via the strong electrostatic binding between the polyanionic heparin motif on the targeting component and the poly-cationic CPP on the drug component. Both systems possessed dual active- (via the antibody or an external magnetic force) and passive- (via the EPR effect [31,52]) targeting functions as well as the potent CPP-mediated cell-internalization features. Once the prodrug complexes were being accumulated at the tumor site, the CPP-linked drug conjugates were released by the administration of protamine, a clinical heparin antidote that possessed a stronger binding affinity to heparin than the CPP). Following their detachment from the targeting component, the CPP-linked drug conjugate would internalize tumor cells, leading to potentially a non-invasive, clinically-enabled yet safe treatment of the tumors (see Figure 3).

By adopting the delivery strategy described in Figure 3a which was termed the “ATTEMPTS” system and developed by us [17], gelonin, a toxin that inhibited protein synthesis through the cleavage of the single adenine residue in ribosomal RNA [100], was delivered for colorectal cancer therapy. In practice, a chimeric TAT-gelonin (TAT-Gel) fusion protein was first genetically produced [101]. A conjugate consisting of a heparin molecule and a murine anti-CEA antibody T84.66 (T84.66-Hep) was also chemically synthesized. These two protein conjugates were assembled together through an electrostatic interaction between heparin and CPP to formulate the prodrug protein complexes. In vivo investigation on animals harbored with the CEA over-expressed LS174T colorectal cancer cells revealed a strong inhibition on tumor growth by these protein complexes, with markedly reduced side-effects on normal tissues. In addition, a selective and significantly augmented (about 58-fold higher) tumor inhibition by the TAT-gelonin/T84.66-Hep complexes was observed in mice, when comparing with the results by using the TAT-gelonin conjugates alone [101]. In another study using this ATTEMPTS system and aspariginase (ASNase) as the protein therapeutics on DBA/2 mice bearing with L5178Y mouse lymphoma cells, we found that the anti-lymphoma treatment could be turned on and off by adding protamine as the triggering agent [17,102]. Animal studies also demonstrated that mice inoculated with L5178Y cells and treated with TAT-ASNase yielded an extended survival rate that was 13% higher than that of the control group [103].

In the instance of the delivery strategy described in Figure 3b, we took the advantages of the transcellular transport ability of MION under the influence of an external magnetic field to establish a therapy/diagnostic-combined system, which recently was widely termed as the “theranostic” system, in achieving simultaneous tumor imaging and drug therapy. Very promising and exciting results were obtained, as we achieved the first experimental success in a rat model of delivering microgram quantity of the very large β-galactosidase protein (MW: > 500 kDa) selectively into a brain tumor but not to the ipsi- or contra-lateral normal brain regions. Since macromolecular therapeutics, such as toxin or siRNA drugs, possessed the ability to fully (>99.9%) eradicate a tumor at the nano-molar dose range, the prospects of achieving a clinically enabled brain tumor drug therapy, based on these findings, suddenly appeared to be practically achievable [104].

### 5.2. The pH-Triggered, CPP-Mediated, “Smart” Intracellular Drug Delivery Systems

Extracellular or intracellular pH values altered significantly in certain specific organs/cellular compartments under pathological situations, such as cancer or inflammation. These subtle environmental pH changes would cause a reversal of charges of the ionizable poly-acids or poly-bases, giving the chance to manipulate the exposure of the CPP moieties on the carrier surface, facilitating the CPP-mediated intracellular drug delivery. Two primary methods were adapted to achieve this pH-responsive drug delivery strategy. First, the aforementioned charge reversal of the poly-acids or poly-bases in response to the environmental pH changes could be employed to induce conformational and/or solubility variations between the polymer and CPPs, obtaining a CPP-mediated intracellular uptake of the drug-loaded carriers. In addition, acid-sensitive linkages such as the benzoic imine and/or the hydrazone bonds could be employed to enable the exposure of the CPP moieties on the carrier surface, triggering an effective cell uptake of the nano-carriers.

**Figure 4:**
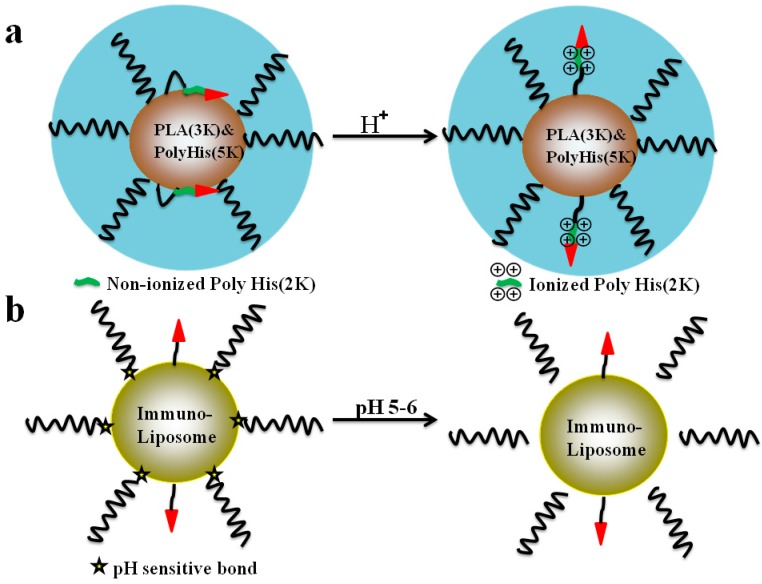
Examples of the pH-sensitive nano-carriers equipped with an efficient CPP exposure: (**a**) polyHis-based micelles responded to acidic tumor microenvironments by an efficient CPP exposure; and (**b**) TAT-peptide-decorated liposomes comprising a hydrolyzable PEG shell allowing improved exposure of the TAT (redrawn according to [105,106]).

As displayed in Figure 4a, the polyhistidine (polyHis)-based micelles (consisting of poly(l-lactic acid) (PLA) (3 kDa)-b-PEG (2 kDa)-b-polyHis (2 kDa)-TAT and polyHis (5 kDa)-b-PEG (3.4 kDa)) was designed to respond to acidic tumor microenvironments in solid tumors, with efficient exposure of the CPPs sequence and further enhanced internalization efficiency of the designed micelles [105]. In this study, the position of shielded CPP is determined by the ionization state of the polyHis (2 kDa) block on the surface of the micelles. At pH about 7.4, CPP was attracted by the interfacial hydrophobic interactions between the nonionized polyHis and PLA core and shielded within the hydrophilic PEG corona shell. However, prior to ionization of the longer polyHis (5 kDa) polyHis (2 kDa) became ionized at extracellular pH (pHe) and exposed CPP on their surface, leading to facilitate the internalization process of the micelles [105]. By this way, the doxorubicin (DOX) potency was greatly increased in both wild and multidrug resistant cell lines (the IC_50_ values were 3.8–8.8 times lower than that of free DOX) [105].

In order to enhance the intracellular delivery capabilities of the engineered nano-carriers to the targeted site, the “ultimate” CPP-functioned carrier containing pH-sensitive linkages such as the benzoic imine and/or the hydrazone bonds demonstrated substantial promise. Briefly, these systems possessed prolonged circulation in the blood, specific cell-surface recognition moieties and acid-response capabilities at the pathological site characteristic either to expose the “hidden” active moieties and/or release an entrapped drug by the surface-attached pH-sensitive coatings [106]. For example, as shown in Figure 4b, a multifunctional immune-liposome decorated with the nucleosome-specific antibody mAb 2C5, was prepared containing CPP moieties which was sterically shielded by in PEG via a pH-degradable hydrazone bond between the PEG chains and PE. At normal pH, the CPP moieties were “shielded” by the long PEG blocks on the surface of the immune-liposomes; once exposed to lowered pH, the hydrazone bond was degraded and removal of the long chain PEG blocks. In turn, the cell penetrating capabilities through the cell membrane of the carriers was enhanced by the liberated CPP moieties [106]. In addition, some other strategies for the pH-responsible delivery strategy involved the exposure of the CPPs by hydrolysis of benzoic imine [107], or even by the protonation of titratable ligand-functionalized lipids with consequent lipid-bilayer reorganization [108]. In other words, cell internalization of the CPP modified carriers could be promoted by the exposure of CPPs caused by surface-charge reversal of the polymer from negative or neutral to positive under acidic pH at tumor tissues [109], or the hydrolysis of the linkages, and this strategy seemed reasonable. However, in the case of in vitro cell culture models, it is likely that the degree of pH-sensitive linkage cleavage or the charge reverse would decrease as extracellular pH was higher than that of the pH at tumor site in animal models.

### 5.3. Enzyme Triggered, CPP-Mediated “Smart” Drug Delivery Systems

**Figure 5:**
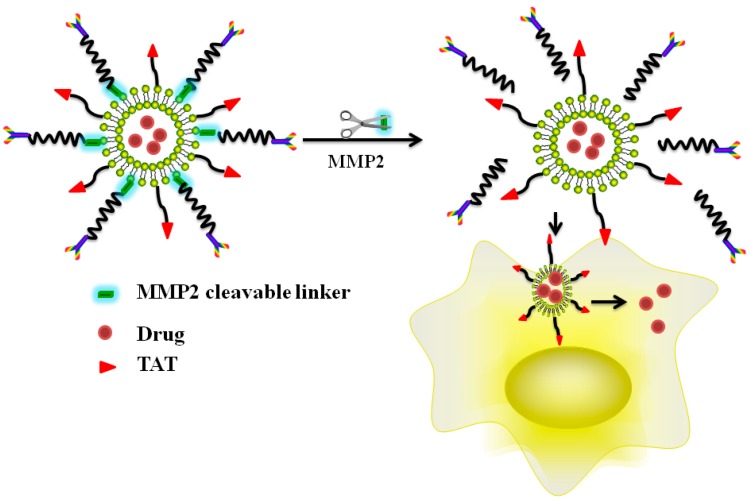
Illustration of the MMP2-triggered, CPP-mediated intracellular delivery strategy of drug-encapsulated liposome (Reproduced according to [114]).

Under pathological conditions, such as in the case of cancer or inflammation, the expression levels of certain tumor surface proteases, such as phospoholipases or glycosidases, altered significantly from those in normal tissues. This variation was exploited to design an enzyme-triggered drug release strategy after successful accumulation of the drug cargos at the tumor target [110]. Several recent studies reported the development of MMP-triggered intracellular delivery strategy by utilizing CPP-functionalized liposomes [111] or dextran-coated MION carriers [112] (Figure 5). These strategies all involved the use of MMP-specific peptide sequences as linkers between the surface of the carrier and PEG chains. Once accumulated in the microenvironment of the tumor target, the PEG shell on the carrier surface would be detached by the action of tumor MMPs, allowing the exposure of the CPP moieties and thus triggering their potent cell penetration functions [112]. Among such systems, the protease-responsive liposomes and nano-carriers containing therapeutic genes or siRNA agents have drawn wide attention. By using this strategy, an almost 70% gene-silencing activity in mice was observed following systemic administration of the siRNA-loaded nanoparticles [113]. Indeed, this tumor-activatable intercellular delivery strategy has been considered as a promising tool in achieving highly effective yet least toxic gene therapy. In spite of all these progress, however, more efforts are still essential to fine-tune this system for possible clinical applications, such as the precise enzyme levels on the tumor surface, cell uptake efficiency, and the in vivo correlation of drug release data with tumor enzyme activities are all needed to be addressed [114].

## 6. Conclusions and Outlooks

The ability to target macromolecular drugs to specific cells and sub-cellular components has a great impact on achieving the CPP-mediated biomedical applications. While a host of exciting and innovative CPP-based drug delivery systems has been established in recent years, the success in delivering protein- or nucleic acid-based drugs to tumor targets and controlling the trajectory within the cells still remains relatively ordinary and primitive. In addition, quantitative assessment of the pharmacokinetics, tumor-available drug concentrations, delivery mechanisms, as well as uptake and trafficking of the CPP-mediated delivery is still insufficient or lacking, resulting in the uncertainty in predicting the therapeutic efficacy and drug-induced toxic effects of the designed drug delivery systems. Furthermore, the aforementioned barriers and limitations would also whittle down the benefit of CPPs, hampering the clinical utility of these promising CPP-mediated intracellular delivery strategies. To this regard, fundamental understanding of the mechanisms, cell uptake trajectories, endosome escape tactic, cell trafficking event and cellular responses deem to be essential, aside from issues of how these smartly engineered, CPP-based delivery systems interact with organelles and cellular components under physiological and/or pathological conditions [114].

Overall, an optimally designed CPP-based “smart” delivery platform should function in the way that during circulation and targeting, the CPP moieties attached on the carrier surface would be sterically shielded from exposure, thereby avoiding the non-specific interaction of the CPP Trojan horse with normal tissues. Once accumulated at the tumor target via either passive or active targeting, protection on these CPP moieties would be removed automatically via the triggering by local environmental stimulus of the tumor, rendering them to be exposed to the tumor tissues and thus promoting the penetration and delivery of the drug payload into cell cytosol. More specifically, in order to realize effective in vivo applications, the engineered CPP-based “smart” delivery systems should be able to overcome many physiological barriers before being taken into the targeted cells, such as avoiding undesirable host immune responses, retaining its colloidal stability during blood circulation, evading extravasation from the targeted tissues, and attaining the most effective delivery into targeted cells [114]. Moreover, unique patho/physiological conditions of the tumor should be creatively applied to initiate a site-specific uptake of the drug payload, thereby offsetting the overwhelming drug-induced toxic effects on normal tissues.
